# Assessing the spatiotemporal interactions of mesopredators in Sumatra’s tropical rainforest

**DOI:** 10.1371/journal.pone.0202876

**Published:** 2018-09-19

**Authors:** Iding Achmad Haidir, David Whyte Macdonald, Matthew Linkie

**Affiliations:** 1 Indonesian Ministry of Environment and Forestry, Jakarta, Indonesia; 2 Wildlife Conservation Research Unit (WildCRU), Department of Zoology, University of Oxford, The Recanati-Kaplan Centre, Tubney, United Kingdom; 3 Wildlife Conservation Society, Bogor, Indonesia; Auburn University, UNITED STATES

## Abstract

Co-occurrence between mesopredators can be achieved by differentiation of prey, temporal activity, and spatial habitat use. The study of mesopredator interactions is a growing area of research in tropical forests and shedding new light on inter-guild competition between threatened vertebrate species that were previously little understood. Here, we investigate sympatry between the Sunda clouded leopard (*Neofelis diardi*) and Asiatic golden cat (*Pardofelis temminckii*) living in the Sumatran rainforests of Indonesia. We investigate: i) spatial overlap of predator-prey species using a combination of single-species occupancy modelling and Bayesian two-species modelling, while controlling for the possible influence of several confounding landscape variables; and, ii) temporal overlap between mesopredators and their shared prey through calculating their kernel density estimate associations. From four study areas, representing lowland, hill, sub-montane and montane forest, 28,404 camera trap nights were sampled. Clouded leopard and golden cat were respectively detected in 24.3% and 22.6% of the 292 sampling sites (camera stations) and co-occurred in 29.6% of the sites where they were detected. Golden cat occupancy was highest in the study area where clouded leopard occupancy was lowest and conversely lowest in the study area where clouded leopard occupancy was highest. However, our fine-scale (camera trap site) analyses found no evidence of avoidance between these two felid species. While both mesopredators exhibited highest spatial overlap with the larger-bodied prey species, temporal niche separation was also found. Clouded leopard was more nocturnal and, consequently, had higher temporal overlap with the more nocturnal prey species, such as porcupine and mouse deer, whereas the more diurnal golden cat had higher overlap with the strictly diurnal great argus pheasant. The Bayesian two species occupancy modelling approach applied in our study fills several important knowledge gaps of Sumatra’s lesser known mesopredators and provides a replicable methodology for studying interspecific competition for other small-medium sized carnivore species in the tropics.

## Introduction

Co-occurrence within a predator guild can be achieved through the differentiation of prey base composition, segregating temporal activity, and segregating spatial overlap within habitat patches [1, 2]. For example, spotted hyaenas *Crocuta crocuta* segregate their temporal movement patterns from the apex predator, lion *Panthera leo*, in order to reduce the likelihood of direct confrontations [3]. When there is direct interaction in resource-rich patches, such as water holes, prairies and open grasslands, competing predators may become less tolerant and more aggressive [4]. Within carnivores community, habitat differentially would be expected by the mesopredator in response to the habitat use of the apex predator, such as leopards (*Panthera pardus*) foraging in forest patches with lower prey abundance in order to avoid tigers (*Panthera tigris*) that dominate prey-rich areas [5]. These behavioural mechanisms have been demonstrated to promote such co-existence in a variety of felid communities [6, 7].

In Asia, the majority of terrestrial carnivore species are at risk of extinction. Understanding their population status and inter-guild interactions is therefore important for conservation managers, especially when the species in competition are both threatened [8]. Most studies on predator interactions in Asia have focussed on the high profile conservation flagship species of tiger and leopard and most have come from the dry deciduous forests of South Asia [6, 8]. Thus, little attention has been paid to the mesopredators, such as the smaller felids species living in Southeast Asia’s humid evergreen rainforest, which are also under threat, lack data but are presumed to be in need of active management.

Existing in-depth references for clouded leopard and golden cat, and other mesopredator, spatiotemporal interactions from Sumatra are scarce. In other parts of the world however, research on these interactions amongst mesopredators and also with their prey is growing. A camera trap study on multi-species occupancies in three national parks from northern Pakistan, revealed that the Altai mountain weasel *Mustela altaica* is associated with its prey, the pika *Ochotona dauurica*, and segregates spatiotemporal activity with red fox *Vulves vulves* and stone marten *Martes fiona* [9]. Moreover, a combined survey of camera trap, distance sampling and faecal analyses is advancing understanding of tiger and leopard density as well as shifting temporal and spatial patterns of both species over time and areas [8].

In this study we investigate interspecific competition between two threatened felid species, the Near Threatened (NT) golden cat (*Pardofelis temminckii*) [10] and the Vulnerable Sunda clouded leopard (*Neofelis diardi*) [11] from the rainforests of Sumatra, Indonesia. These species have similar body sizes, semi-arboreal behaviour and prey base, therefore neither species is obviously dominant. Previously, Linkie and Ridout [12] investigated the temporal overlap of clouded leopard, golden cat and tiger (*Panthera tigris*) using a camera trap-based sampling technique in multiple study areas that revealed temporal separation to be 10–20% greater between the two smaller felid species. A comparison using single-species, single-season occupancy models then revealed that clouded leopard tended to use forest at higher elevation and further from the non-forest edge, whereas golden cat preferred lower elevation forest with no such edge effect, thereby suggesting the presence of spatial separation [13]. However, these two preliminary studies did not consider how interspecific competition was influenced by prey availability and activity patterns in space and time, nor did they explicitly test spatial interactions between the two carnivore species. Thus, fundamentally important ecological questions remain unanswered, such as whether predator species occupancy is more strongly influenced either by competitor co-occurrence, by prey availability or indeed abiotic landscape factors.

Other carnivore species that might compete with the clouded leopard and golden cat include Sumatran tiger, Asiatic wild dog (*Cuon alpinus*) and sun bear (*Helarctos malayanus*). However, it is unlikely that these three species are true competitors with the two medium-sized felids for several reasons. Tiger and dhole are strictly ground dwelling [2] and the pack-hunting dhole is able to hunt larger-sized prey, such as adult sambar [14, 15], which would be difficult to capture, at least as adults, for the smaller solitary felids. The sun bear is omnivorous with a diet largely consisting of fruits and invertebrates, rather than ungulate prey [16]. The remaining, also solitary, Sumatran felid species are marbled cat (*Pardofelis marmorata*), leopard cat (*Prionailiurus bengalensis*) and flat-headed cat (*Prionailurus planiceps*). The average body size for each species is approximately 50 per cent smaller than golden cat and clouded leopard which makes them unlikely competitors.

To investigate interspecific competition, we use a combination of single-species occupancy modelling and Bayesian two-species occupancy modelling with parameterization, which tests for species dominance as a proxy for co-existence [17]. From this, we measure the potential of predator-prey interactions due to spatial overlap, while analytically controlling for the possible influence of confounding landscape factors. Once we have understood the spatial relationship between these cats, we then calculate kernel densities for the two predator species and their shared prey species to explore overlap in activity patterns. More specifically, this study investitages: 1) level of spatial overlap of clouded leopard and golden cat using a combination of single-species occupancy modelling and Bayesian two-species modelling that incorporate controlled landscape factors; and, 2) measure clouded leopard and golden cat activity patterns; given that their occurrences and activity patterns are affected by shared potential prey.

## Materials and methods

### Study area

The study was conducted in Kerinci Seblat National Park and its adjacent forest that are under State land authority. We have secured permit to conduct the fieldwork from Indonesia Ministry of Environment and Forestry (MoEF) technical unit in Jambi, West Sumatra, South Sumatra and Bengkulu, of whom the first author is an employee of MoEF.

**Fig 1:**
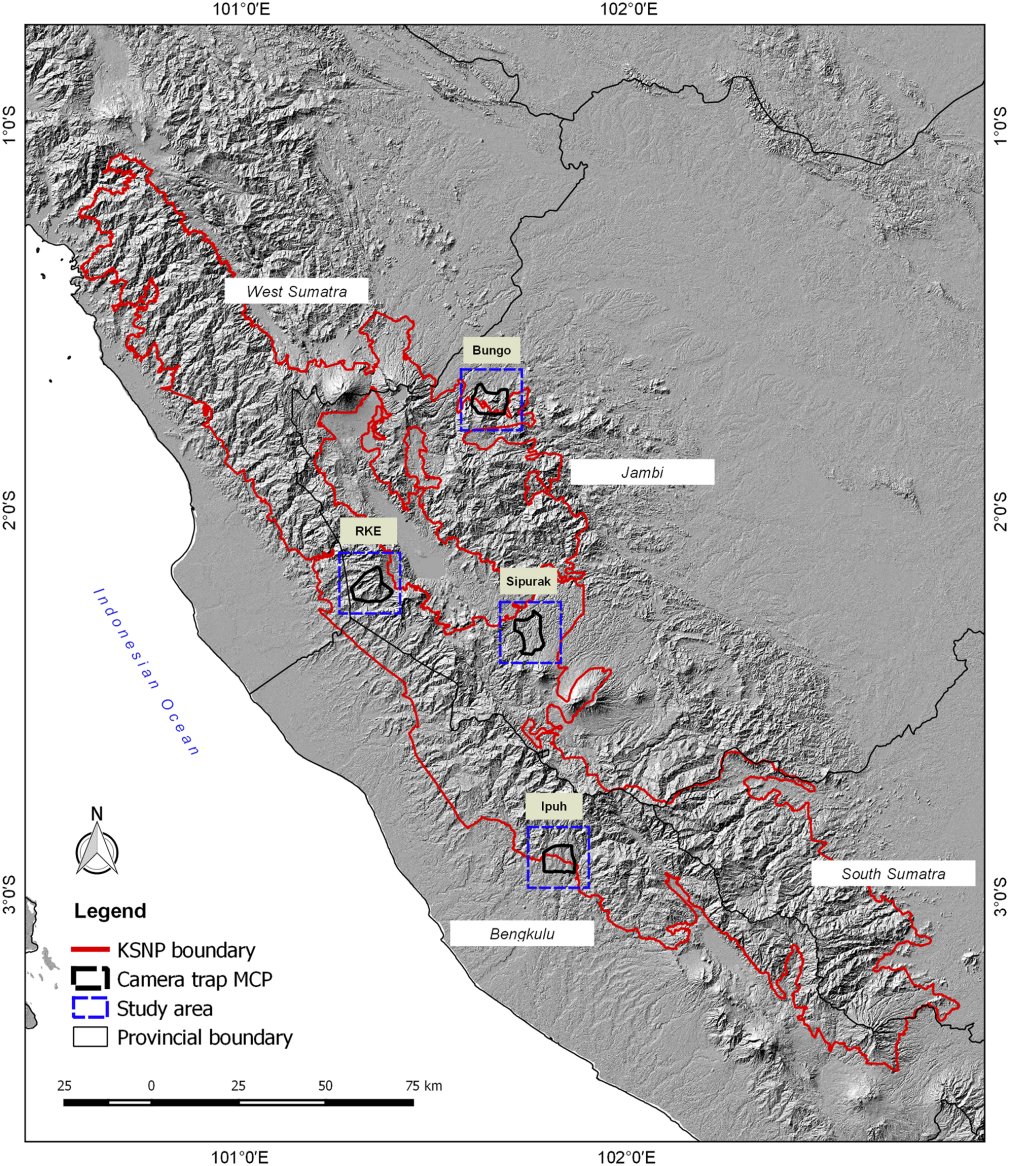
Location of the four-rainforest study areas, of varying elevation in and around Kerinci Seblat National Park, west-central Sumatra.

**Fig 2:**
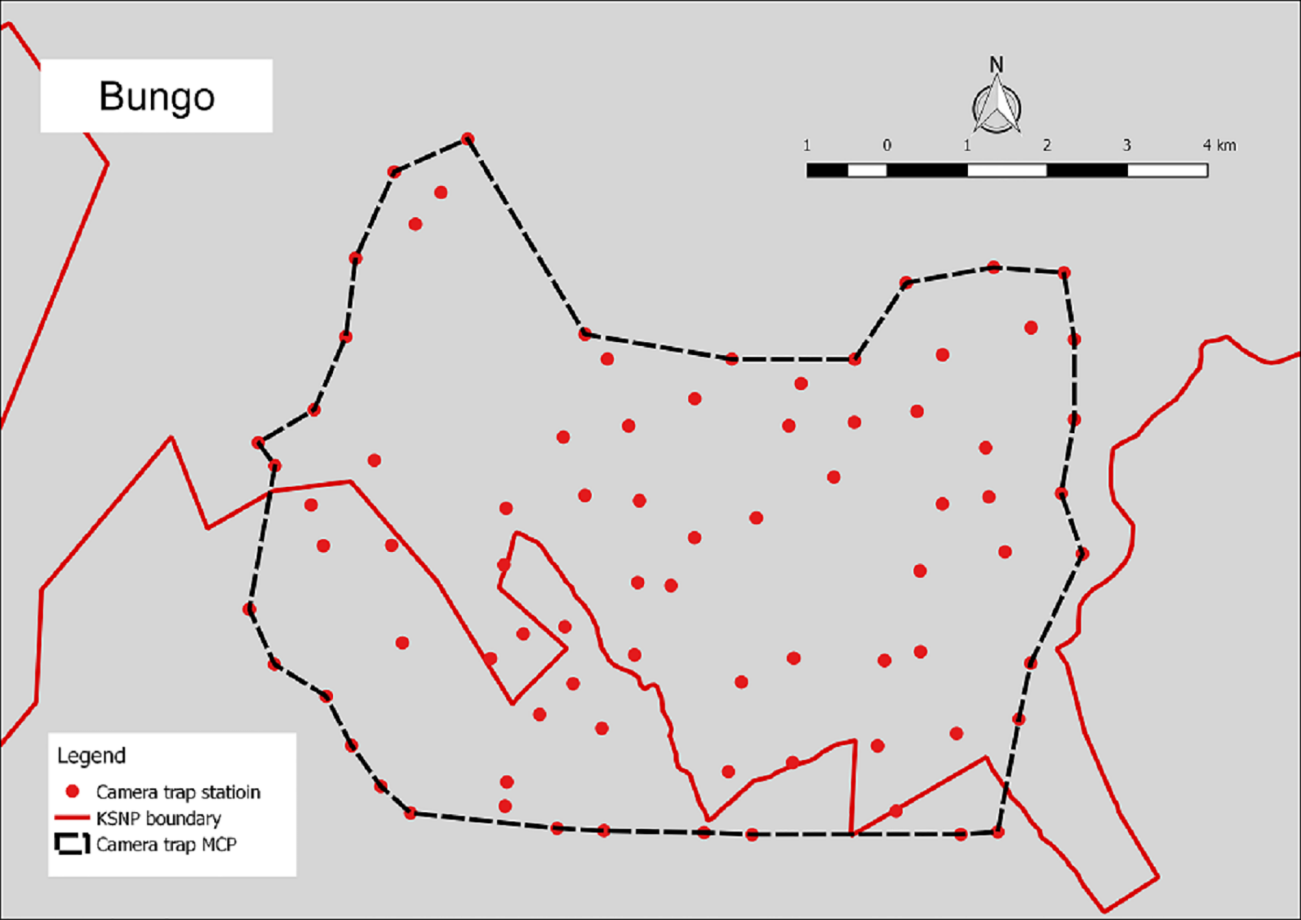
Camera trap location in Bungo.

**Fig 3:**
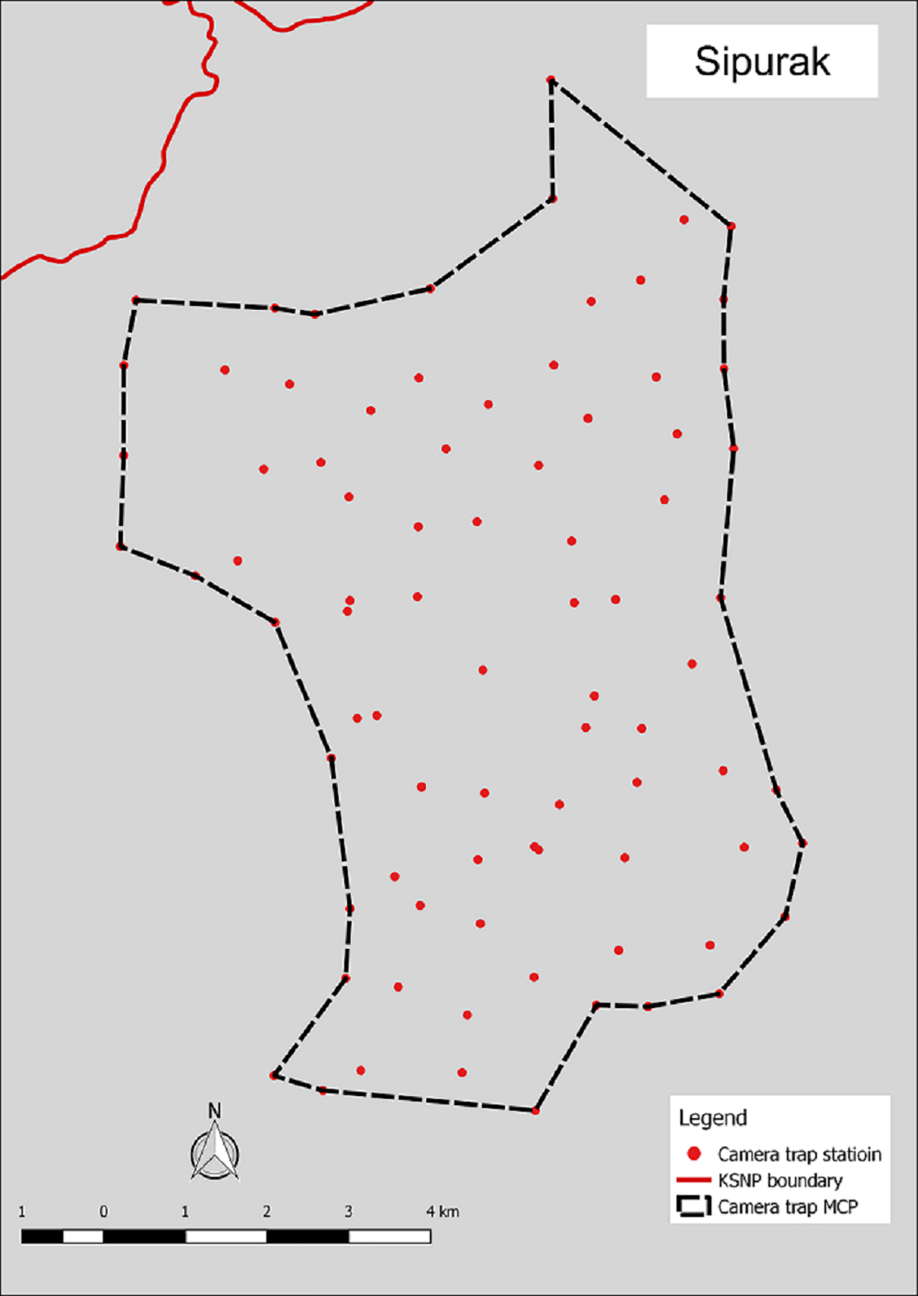
Camera trap location in Sipurak.

**Fig 4:**
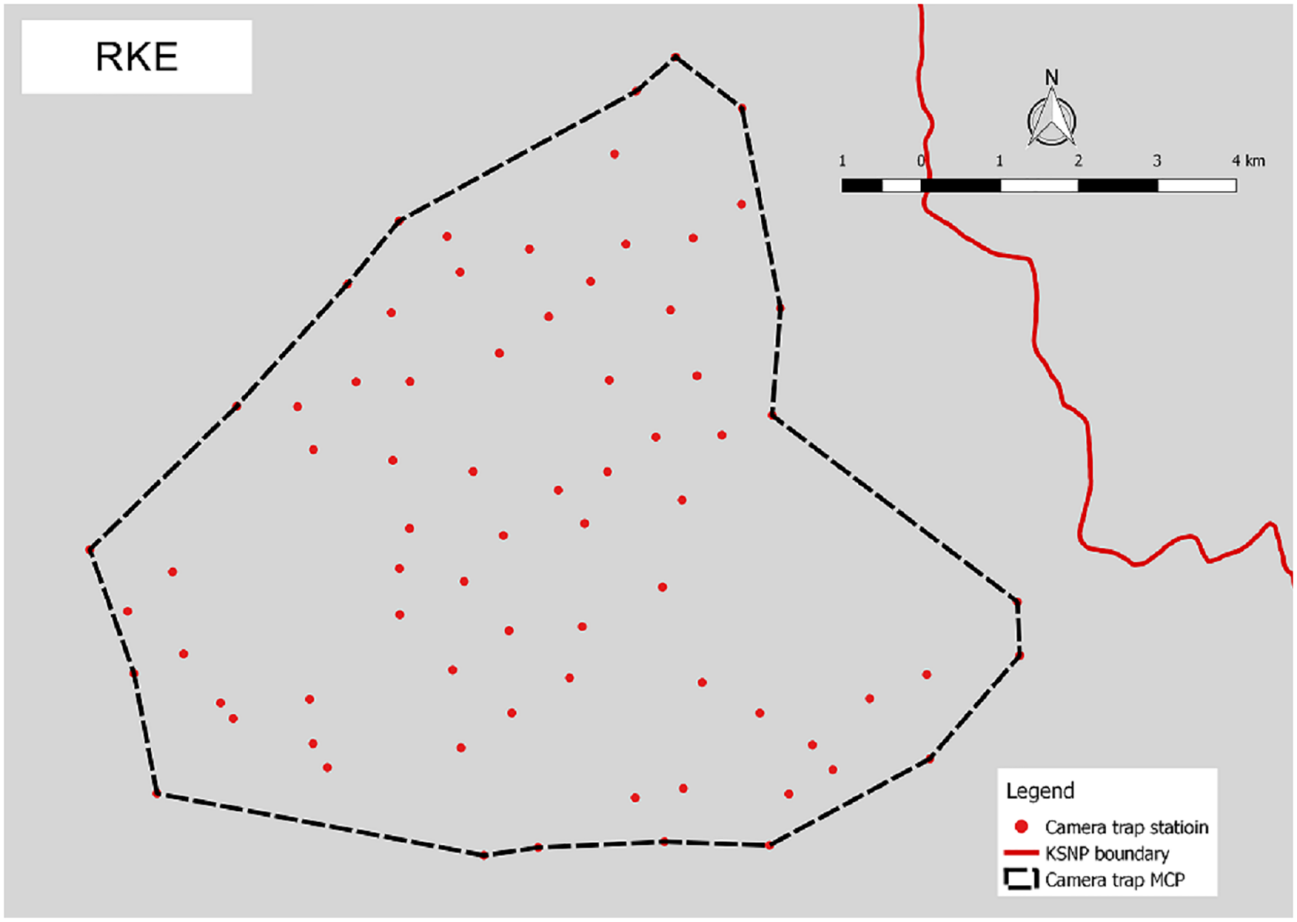
Camera trap location in Renah Kayu Embun (RKE).

**Fig 5:**
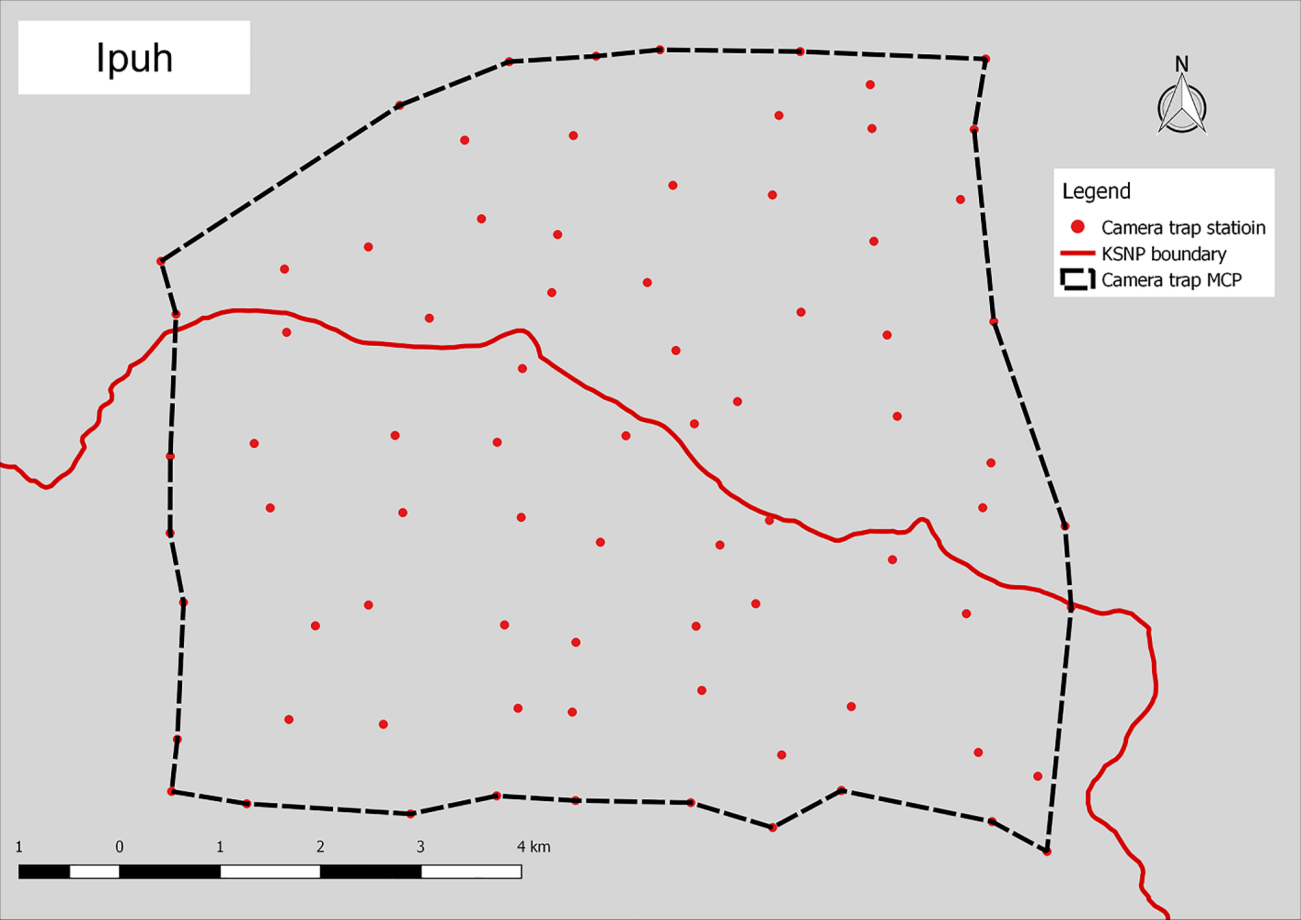
Camera trap location in Ipuh.

Camera trap surveys were conducted in four humid evergreen rainforest study areas located in west-central Sumatra, Indonesia. The ~26,000 km^2^ Kerinci Seblat landscape lies between E 100° 35’ 00” and E 102° 54’ 00”, and S 1° 30’ 00” and S 3° 37’ 00”. Two areas were situated entirely inside the 13,900 km^2^ Kerinci Seblat National Park, whereas the other two areas straddled the park border (Figs 1–5, Table 1). The four study areas represent the main forest types in the landscape (lowland, hill, sub-montane and montane), which are characterized by their varying elevation. The national park stretches for ~370 km from north to south and 40–70 km from east to west. The park is situated on the central spine of the Bukit Barisan mountain range and spans elevations of 250 to 3,805 m asl (above sea level), the peak of Mount Kerinci. The average monthly rainfall is high, only dropping below 1500 mm during the months of July and August [18].

**Table 1:** Camera trap study area characteristics and sampling effort.

Feature	Study area			
	Bungo	Sipurak	RKE	Ipuh
Mean elevation (min.—max.) in metres	630 (310–1,120)	800 (370–1,070)	1,190 (490–2,000)	430 (230–670)
Main forest types	Hill–submontane	Hill–submontane	Hill–montane	Lowland–hill
Minimum convex polygon (km^2^)	63.9	62.5	63.2	60.6
# trap nights	8,399	7,053	6,674	6,278
# paired camera trap stations	76	76	65	75
Mean camera trap spacing (m)	777	814	1,027	793
Survey period	June-Nov 2014	Nov 2014-Mar 2015	Apr-Aug 2015	Sept-Dec 2015

### Field data collection

For each of the four study areas, 80 paired camera trap placements were set across 1 km^2^ grid cells that formed a ~60 km^2^ sampling area. Cameras were set at a height of 30–40 cm off the ground, a distance of 2–3 meters from the target trail and at a spacing of 0.8–1.0 km between paired traps. Two brands of heat-motion sensor camera traps were used: Cuddeback Ambush IR (Non Typical Inc., WI, USA) and Panthera IV (Panthera Foundation). Each pair consisted of the same brand at each station, without bait or other lure. Cameras were set by the side of animal trails as indicated by the presence of focal species signs. Camera traps collected data in the field continuously for three months and were visited every two weeks for maintenance and data retrieval.

Understanding interactions amongst mesopredators requires knowledge of their diets. In Southeast Asia, there are only a few published dietary studies on clouded leopard and golden cat. From Thailand, these felids were found to consume prey as small as Muridae (0.1 kg) and as big as Indian hog deer (*Axis porcinus*, ~40 kg) for clouded leopard and muntjac (*Muntiacus muntjac*, ~30 kg) for golden cat [19]. From Malaysia, golden cat was found to hunt mouse deer (*Tragulus* spp., ~4.5 kg), Muridae (~0.2 kg) and dusky leaf monkey (*Trachypithecus obscurus*, ~6.5 kg) [20]. A record from Borneo confirmed that clouded leopard attacked both pig-tailed macaque (*Macaca nemestrina*, 5–15 kg) and long-tailed macaque (*M*. *fascicularis*, 4–8 kg) [21]. In Sumatra, golden cat have been recorded attacking poultry [22]. Based on these records and the known prey from our study area, we selected five species as potentially preferred prey: muntjac (*M*. *muntjac* and *M*. *montanus*), mouse deer, pig-tailed macaque, porcupine (*Hystrix brachyura*) and great argus pheasant (*Argusianus argus*).

#### Spatial data analysis

For each camera trap placement, information on the landscape covariates of elevation, distance to forest edge and distance to river were generated using a GIS. These variables were chosen because they have been found to have an effect on felid distribution elsewhere in Sumatra [22, 23]. Elevation data, at 90 m resolution, were obtained from the Shuttle Radar Topography Mission [24]. River data were obtained from the Indonesian National Coordination Agency for Surveys and Mapping for Sumatra (UTM 47 S, scale 1:50,000). Forest edge data, converted to shapefile format, were obtained from a forest cover map that was produced in 2013 [25].

To investigate species spatial occurrence, an occupancy-modelling framework based on the following assumptions was used: i) demographic population closure—sites have a constant species occupancy status over the sampling period (i.e. there are no births, deaths, immigrations or emigrations); ii) sites and replicates (occasions) are spatially and temporally independent; and, iii) the influence of species detectability and spatial sampling are accounted for in the models [26]. Individual detection matrices (‘1’ = detected and ‘0’ = undetected) were constructed for the focal species using 14 day sampling occasions [13]. Clouded leopard and golden cat gestation period is 80–90 days [27], in order to anticipate that there would have been new births during our study we recorded only adult individuals in our detection matrices within ~100 days of survey.

A Bayesian multi-species occupancy-modelling framework that enables the modelling of interactions between paired species was used to assess spatial associations between clouded leopard and golden cat and then individually with each of the prey species [17]. Four co-occupancy scenarios were considered: i) golden cat occupancy is influenced by the occupancy of the slightly larger clouded leopard; ii) clouded leopard occupancy is influenced by prey occupancy; iii) golden cat occupancy is influenced by prey occupancy; and, iv) prey and predator occupancies are both influenced by site covariates.

For the multi-species occupancy modelling, we used Bayesian 95% credible density intervals (CDI) for the mean of the posterior distribution for parameter estimates to make inferences about effects on the occupancy of predator species [3, 9]. We used packages R2jags and rjags in R statistical software to run the multi-species occupancy models [28, 29]. Uninformative-uniform priors defined by the log-odds interval [–10,10] for all parameter distributions with four chains of 50,000 iterations each, including 10,000 iteration burn-ins, were run. Based on the calculation, we assessed model convergence using the $\hat{R}$ value (closer to 1.0 indicating a more plausible convergence) and from a visual inspection of chain trace plots [30].

#### Overlap in activity pattern

To investigate temporal interactions between the two felid species and then paired individually with each prey species, study area data were separately analysed to investigate differences in species temporal pattern. Independent photographs were treated as a random sample from the underlying distribution that describes the probability of a photograph being taken within any particular interval of the day [12, 31].

To analyse species interaction data from camera trapping, we follow Ridout and Linkie [31] that used Kernel Density Estimate (KDE) for finite data smoothing with three delta estimators.

## Results

### Species detection

From 292 paired-camera trap stations, 39 vertebrate species from 20 families were recorded over 28,404 trap nights from the four study areas (S1 Table). Combined data from four study areas included independent photographs of clouded leopard (n = 163), golden cat (107), macaque (1,215), great argus pheasant (1,090), muntjac (803), porcupine (513), and mouse deer (189).

### Spatial occurrence patterns

From the combined camera trap data set, clouded leopard was detected at 71/292 stations, yielding a naïve occupancy (percent of occurrence) estimate of 0.24, whereas golden cat was detected at 66/292 stations (0.23). For the focal prey species, naïve occupancy estimates were recorded for pig-tailed macaque (0.73), muntjac (0.63), great argus pheasant (0.47), porcupine (0.38) and mouse deer (0.19), but these varied between study areas. In the individual study areas, clouded leopard occupancy was highest in RKE (0.57) and lowest in Ipuh (0.26) with varying study area detection probabilities ($\hat{p}$ = 0.09–0.23; Table 2). Golden cat occupancy was highest (0.53) in Sipurak where clouded leopard occupancy was lowest, but was lowest (0.39) in RKE where clouded leopard occupancy was highest (Table 2). Both species had low occurrence in the Ipuh study area. See S2 Table for details on the occupancy models for each species.

**Table 2:** Single season-single species occupancy estimates ($\hat{\psi }$), detection probability (p) and associated landscape covariates for two other predator species and their potential prey.

Species/Study Area	$\hat{\psi }$ (95%CI)	$\hat{p}$ (95%CI)	β Coefficient (95%CI)		
			*Elevation*	*Dist*. *to river*	*Dist*. *to forest edge*
*Clouded leopard*					
Bungo	0.49	0.09	-0.57	-0.70	-
	(0.22–0.76)	(0.04–0.17)	(-1.22)-(-0.20)	((-1.31)-(-0.77))	
Sipurak	0.26	0.12	-	-	-
	(0.12–0.49)	(0.05–0.24)			
RKE	0.56	0.20	-	1.20	1.50
	(0.37–0.74)	(0.14–0.29)		(0.21–2.18)	(0.44–2.57)
Ipuh	0.25	0.23	-	-	-
	(0.16–0.39)	(0.15–0.34)			
*Golden cat*					
Bungo	0.46	0.06	-	-	-
	(0.12–0.85)	(0.02–0.16)			
Sipurak	0.53	0.12	-	-	-
	(0.29–0.76)	(0.07–0.19)			
RKE	0.39	0.14	-	-	-
	(0.19–0.64)	(0.14–0.25)			
Ipuh	0.39	0.07	-	-	-
	(0.11–0.76)	(0.03–0.20)			
*Muntjac*					
Bungo	0.75	0.30	1.10	1.14	1.31
	(0.62–0.84)	(0.26–0.35)	(0.48–1.73)	(0.52–1.76)	(0.61–2.01)
Sipurak	0.88	0.31	-	-	-
	(0.75–0.95)	(0.27–0.36)			
RKE	0.31	0.22	-1.87	-2.09	-2.06
	(0.18–0.47)	(0.14–0.33)	(-2.93-(-0.80)	(-2.94)-(-1.24)	(-2.91)-(-1.21)
Ipuh	0.74	0.24	-	-	-
	(0.57–0.86)	(0.19–0.30)			
*Mouse deer*					
Bungo	0.46	0.11	-0.90	-0.90	-0.83
	(0.25–0.68)	(0.06–0.19)	(-1.43 –(-0.37)	(-1.41)-(-0.39)	(-1.37)-(0.29)
Sipurak	0.24	0.16	-	-	-
	(0.13–0.41)	(0.09–0.28)			
RKE	0.04	0.19	-2.44	-2.44	-2.41
	(0.01–0.20)	(0.03–0.62)	(-4.11)-(-0.77)	(-3.55)-(-0.94)	(-3.91)-(-0.90)
Ipuh	0.27	0.34	-	-	-
	(0.18–0.38)	(0.32–0.37)			
*Macaque*					
Bungo	0.71	0.29	-	-	-
	(0.58–0.81)	(0.24–0.34)			
Sipurak	0.89	0.43	-	-	-
	(0.79–0.95)	(0.38–0.47)			
RKE	0.36	0.38	-	-1.34	-1.39
	(0.25–0.49)	(0.35–0.42)		((-2.07)-(-0.60))	((-2.14)-(-0.65))
Ipuh	0.98	0.28	-	-	-
	(0.23–0.99)	(0.23–0.33)			
*Porcupine*					
Bungo	0.53	0.25	-0.36	-	-
	(040–0.65)	(0.20–0.31)	((-0.88)-0.16)		
Sipurak	0.53	0.35	-1.92	-1.03	-
	(0.41–0.64)	(0.29–0.41)	((-2.78)-(-1.06))	((-1.75)-(-0.31))	
RKE	0.26	0.36	0.44	-0.96	-1.21
	(0.17–0.39)	(0.32–0.41)	((-0.55)-1.43)	((-1.71)-(-0.22))	((-2.02)-(-0.41))
Ipuh	0.30	0.21	0.86	0.13	0.81
	(0.18–0.44)	(0.14–0.31)	(0.10–1.63)	((-0.55)-0.81)	(0.04–1.58)
*Argus pheasant*					
Bungo	0.50	0.29	-	-	-
	(0.38–0.62)	(0.24–0.35)			
Sipurak	0.46	0.37	-	1.20	1.33
	(0.34–0.58)	(0.31–0.44)		(0.46–1.95)	(0.47–2.19)
RKE	0.27	0.33	-	-0.93	-0.99
	(0.17–0.40)	(0.23–0.45)		((-1.68)-(-0.17))	((-1.75)-(-0.24))
Ipuh	0.73	0.36	0.84	-	-
	(0.60–0.82)	(0.31–0.42)	(0.05–1.63)		

Note: $\hat{\psi }$ represents occupancy values for the top ranked model from a list of candidate models that incorporate different site covariates for Psi/ ($\hat{\psi }$) and for a constant detection probability ($\hat{p}$), only significant results (do not contain zero) are shown. The β-coefficient is an intercept values of the linear models that indicates the relationship between occupancy values and the covariates derived from the occupancy modelling

Investigating predator-prey co-occurrence revealed that clouded leopard was detected with all prey combined in 22.2% (62/292) of the sampling units. For individual prey species, it overlapped most with macaque (16.4%), then muntjac (12.6%), porcupine (10.6%), great argus pheasant (9.6%) and mouse deer (2.4%). There was a similar overlap pattern for golden cat with all prey combined in 20.8% of the units and individually for macaque (18.8%), muntjac (15.0%), porcupine (13.0%), great argus pheasant (11.6%) and mouse deer (7.5%).

**Fig 6:**
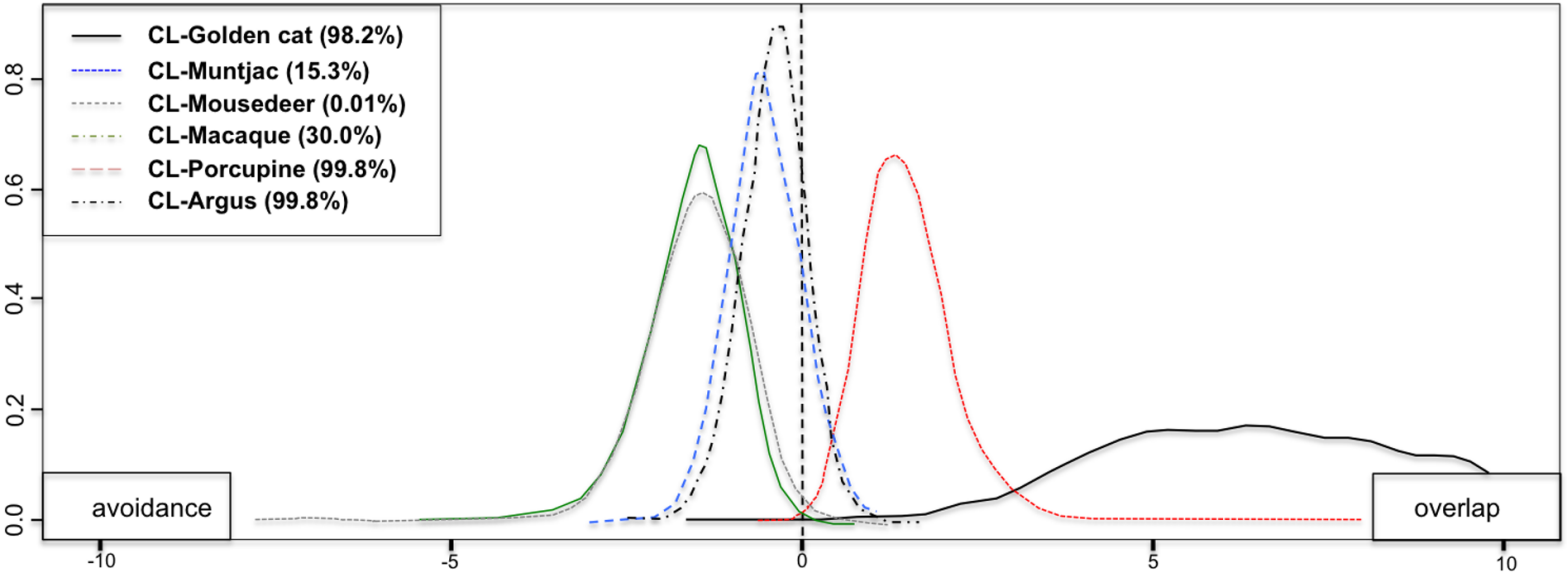
Pair-wise spatial overlap between clouded leopard, golden cat, and prey. Clouded leopard and golden cat (solid black line), and clouded leopard and prey; muntjac (blue dotted line), mouse deer (green dotted line) macaque (blue dashed-dotted line), porcupine (red-dashed line), and argus pheasant (black dashed-dotted line). The right side from straight-dashed line of the graph indicates overlap, whereas the left side indicates avoidance.

**Fig 7:**
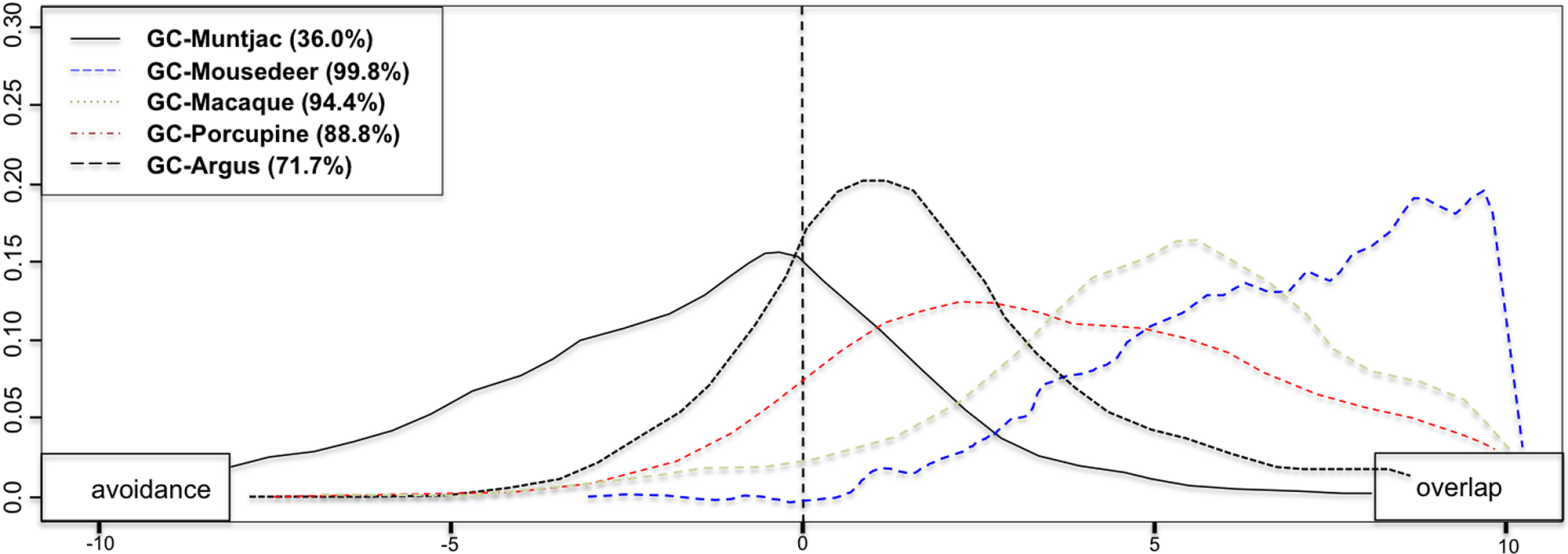
Pair-wise spatial overlap between golden cat and prey. Spatial overlap proportion between golden cat and muntja*c* (black solid line), mouse deer (blue dashed line), macaque (grey dotted line), porcupine (red-dashed line), and argus pheasant (black dashed line). The right side from straight-dashed line of the graph indicates overlap, whereas the left side indicates avoidance.

Spatial co-occurrence between mesopredators and individual prey species, as indicated by beta coefficient (β) and the proportion of spatial overlap (quantity) varied between study areas (Table 3). The multi-species occupancy models revealed that both clouded leopard and golden cat were more likely to use sites at which muntjac and macaque were present, with weak evidence of golden cat occupancy being influenced by clouded leopard presence. The Bayesian multi-species occupancy calculated log odds values between pairwise species hierarchically to determine relationships between species. The proportions of negative log odds values were found higher for clouded leopard and muntjac, mouse deer, macaque and great argus pheasant indicate avoidance by the prey, where golden cat had higher percentage of positive log odds values, which indicates spatial association (Figs 6 and 7; Table 3 and S2 Table).

**Table 3:** Spatial co-occurrence between clouded leopard and golden cat with individual prey species, as indicated by their beta coefficients (β) and the proportion of spatial overlap between study areas.

Species pairs	Bungo	Sipurak	RKE	Ipuh	Overall
	β (% overlap)	β (% overlap)	β (% overlap)	β (% overlap)	β (% overlap)
Clouded leopard+					
golden cat	0.91 (56.7%)	-0.43 (44.9%)	5.47 (89.0%)	2.22 (67.3%)	6.33 (98.2%)
muntjac	2.26 (66.4%)	2.20 (66.2%)	-1.97 (30.8%)	-0.76 (42.8%)	-0.59 (15.3%)
mousedeer	-2.35 (32.2%)	0.35 (52.6%)	-4.42 (16.8%)	-4.88 (6.0%)	-1.59 (0.1%)
porcupine	3.35 (75.4%)	4.27 (86.0%)	7.28 (98.5%)	7.92 (99.6%)	1.63 (99.8%)
macaque	-2.59 (29.0%)	-1.98 (34.4%)	4.29 (84.1%)	-2.9 (27.2%)	-1.75 (1.4%)
argus	1.81 (62.8%)	-2.37 (29.1%)	-4.24 (13.8%)	2.03 (74.5%)	-0.23 (21.6%)
Golden cat+					
muntjac	0.21 (50.8%)	1.35 (61.6%)	-2.45 (30.1%)	1.87 (64.4%)	-2.14 (36.0%)
mousedeer	0.32 (51.8%)	-4.26 (16.9%)	-1.24 (40.7%)	4.62 (85.0%)	4.87 (99.8%)
porcupine	4.20 (83.8%)	7.73 (99.0%)	2.39 (68.3%)	3.07 (73.5%)	4.71 (88.8%)
macaque	4.84 (85.5%)	0.03 (51.5%)	5.53 (91.3%)	0.75 (56.0%)	4.87 (94.4%)
argus	1.98 (65.8%)	5.20 (93.2%)	0.72 (55.2%)	-0.99 (41.0%)	1.71 (71.7%)

Note: Beta coefficient β (range from 1–10 as stated in the model calculation) indicates the degree of positive (+) interaction (overlap) or negative (-) interaction (avoidance), and the percentage of overlap based on log-odds effects from Bayesian hierarchical multi-species occupancy. Grey cells indicate >75% of overlap or avoidance.

### Temporal patterns

**Fig 8:**
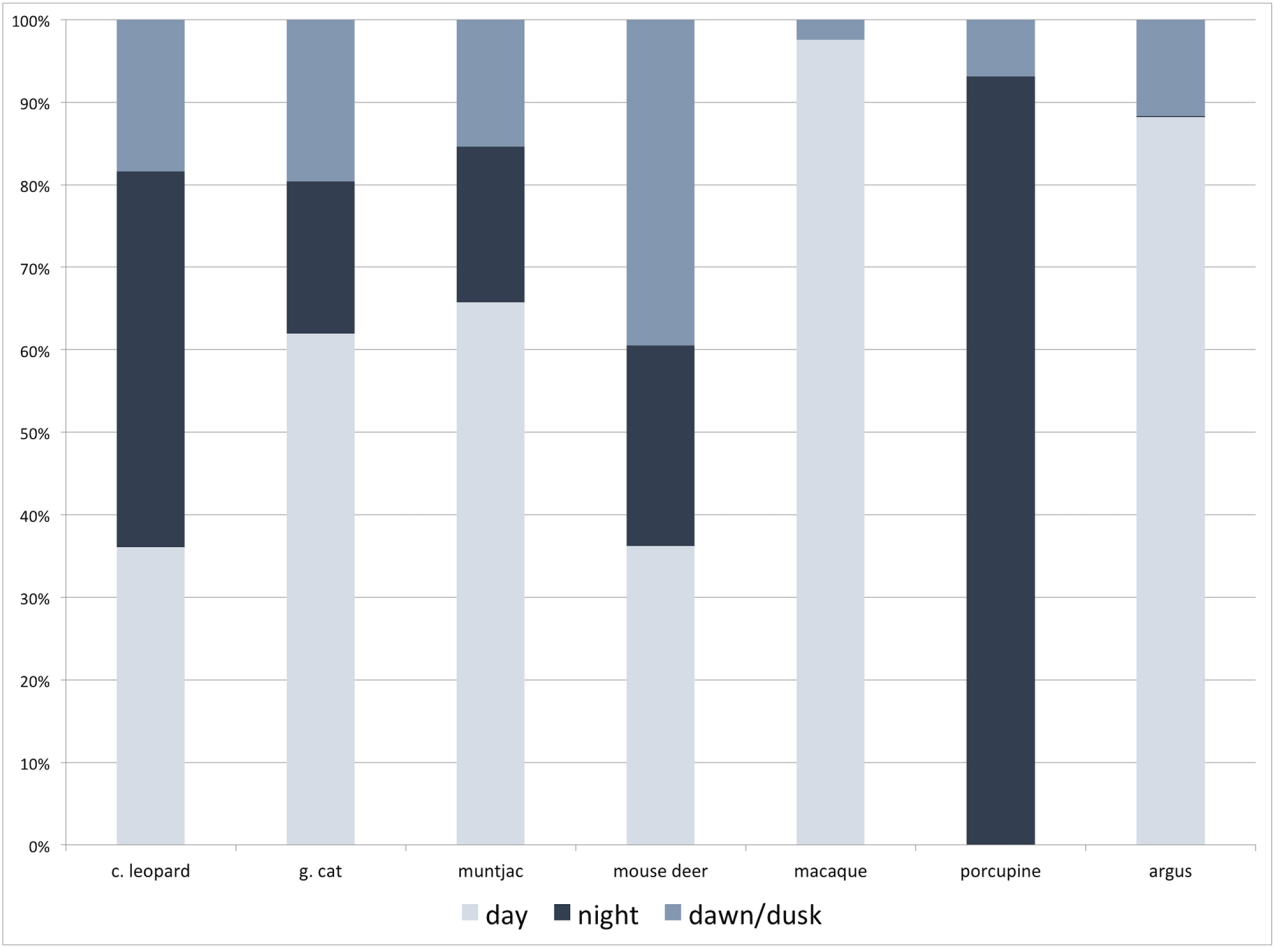
Temporal patterns of clouded leopard (c. leopard), golden cat (g. cat) and their prey. Temporal categorizations for diurnal (06:30–17:30hrs), nocturnal (19:00–05:00hrs) and crepuscular (05:00–06:30hrs and 17:30–19:00hrs).

Combined camera trap data from all four study areas, revealed that golden cat was more diurnal (61.9% of observations between 6:30–17:30hrs) than clouded leopard (36.1%, Fig 8). For the prey species, strongly diurnal patterns were shown by macaque (97.5%) and great argus pheasant (88.2%), and followed by muntjac (65.7%) while porcupine exhibited strongly nocturnal patterns with 93.2% of detections being at night and followed by mouse deer at 63.5%), (19.00–05.00hrs).

Comparing between study areas, the temporal overlap between clouded leopard and golden cat showed little variation, being lowest in Sipurak (Δ_4_ = 0.58) and highest in Bungo and Ipuh (Δ_4_ = 0.62). The predator-prey temporal patterns revealed that clouded leopard, although with some variations across study areas, had the highest overlap with muntjac and mouse deer than with the more diurnal macaque and great argus pheasant. Similarly golden cat had the highest temporal overlap with muntjac but second to that is the more diurnal macaque and great argus pheasant followed by the more nocturnal mouse deer (Table 4; S1 Fig).

**Table 4:** Estimates of temporal overlap between clouded leopard and golden cat with their individual prey species, as indicated by their Kernel density ($\hat{D}$) estimates and the proportion of overlap between study areas.

Species	Study area			
	Bungo	Sipurak	RKE	Ipuh
	$\hat{D}$ with 95% CI			
Clouded leopard				
Golden cat	0.62	0.58	0.61	0.62
	0.50–0.83	0.39–0.77	0.45–0.79	0.47–0.77
Muntjac	0.69	0.54	0.69	0.61
	0.58–0.84	0.37–0.72	0.57–0.82	0.50–0.70
Mouse deer	0.74	0.56	0.53	0.68
	0.67–0.93	0.35–0.78	0.35–0.75	0.54–0.82
Macaque	0.47	0.24	0.49	0.33
	0.32–0.61	0.03–0.35	0.34–0.58	0.20–0.38
Porcupine	0.57	0.62	0.48	0.53
	0.42–0.71	0.46–0.80	0.36–0.61	0.35–0.65
Great argus pheasant	0.42	0.34	0.43	0.56
	0.29–0.57	0.12–0.48	0.31–0.53	0.44–0.70
Golden cat				
Muntjac	0.73	0.70	0.66	0.68
	0.62–0.91	0.58–0.85	0.54–0.87	0.59–0.85
Mouse deer	0.61	0.61	0.39	0.58
	0.47–0.80	0.47–0.82	0.19–0.64	0.44–0.73
Macaque	0.72	0.48	0.64	0.56
	0.58–0.92	0.31–0.59	0.48–0.86	0.40–0.75
Porcupine	0.26	0.43	0.26	0.26
	0.10–0.37	0.25–0.55	0.05–0.39	0.10–0.39
Great argus pheasant	0.63	0.61	0.59	0.68
	0.49–0.82	0.49–0.75	0.43–0.83	0.55–0.85

Each cell indicates a density overlap value, with 95% confidence intervals (CIs) below the averaged value. The value of the trigonometric sum of density overlap ($\hat{D}$) between two species within the different study areas are shaded from lighter to darker indicating low to high overlap.

## Discussion

We found differences in the extent to which clouded leopard and golden cat spatially overlapped with different prey species. The two felids exhibited different temporal activity patterns, which may have enabled a greater degree of spatial overlap. Although detailed dietary studies are lacking, we identified potential prey species whose spatial and temporal activity patterns had a higher degree of overlap with that of the predators’ thereby suggesting specific associations.

### Spatial and temporal overlap patterns

Camera trapping revealed weak evidence for niche partitioning between clouded leopard and golden cat. Although there were signs of habitat partitioning with clouded leopards tending to avoid forest edge patches, whilst golden cats did not, the habitat of the two species overlapped extensively in the forest interior. This builds on the earlier work of Haidir et al. (2013) that found clouded leopard occupancy being higher in patches that were further from the forest edge and at higher elevations. Our study also found that the forest edge had a positive influence on clouded leopard occupancy only at higher elevation where prey abundance was lower, such as in the sub-montane study area of RKE.

Clouded leopard and golden cat are likely to concentrate their hunting where prey is abundant and accessible [32]. The reciprocal results for Sipurak and RKE, where the highest occupancy for one felid species occurred alongside the lowest occupancy of the other, and vice versa, suggests that there is a broad level competition between them. Conversely, in Ipuh, both species had low occupancies, despite the presence of a rich and widespread prey base, which is possibly a result of the much higher human disturbance here [33, 34]. Ipuh contains large tracts of lowland forests that are highly accessible and borders an ex-logging concession. The neighbouring forests in Ipuh also had the highest forest degradation rate amongst the four study areas [13, 35].

In Bungo, the two felid species shared similar and relatively high occupancy rates. Tiger densities were almost twice as high in Bungo than in Ipuh. A relatively higher occupancy rate of tiger, clouded leopard and golden cat could be indicative of higher prey biomass and lower threats to felid species that reduce interspecies competition, especially on prey species amongst the three felid species. Additionally, higher occupancy of both clouded leopard and golden cat might be due to higher prey abundance within secondary forests [33, 36] that would result in tiger exerting a lower influence on mesopredators’ co-occurrence. Documenting the influence of larger predators towards smaller ones is difficult in the dense Sumatran rainforest. This is, unlike in more open habitats in India or central African countries, where studies on predator-prey interactions and their preferred diet can be conducted through direct observations [37, 38]. Studying these interactions in dense tropical rainforests is particularly difficult because inferences are drawn solely from the observed patterns that are derived from remote device, such as camera traps. Nevertheless, our camera trap data revealed a distinction in the temporal activity patterns between the more nocturnal clouded leopard and the more diurnal golden cat would have further reduced interference competition between the two species. These results are consistent with the patterns observed by previous studies conducted in Sumatra [23, 39].

Clouded leopards exhibited the closest spatial and temporal associations with muntjac. A study in Borneo on the interactions between felids and primates reported a clouded leopard killing a pig-tailed macaque [40]. Physiologically, clouded leopards have the longest canines, of any felid species relative to their body size and are likely to have evolved to be able to kill larger-bodied prey such as young muntjac, adult mouse deer and great argus pheasant.

In the case of the golden cat, its diet is less certain but it might target mouse deer and argus pheasant. Unlike the clouded leopard’s spatial occurrence with muntjac and mousedeer, golden cat showed a positive association with these species. A positive association between golden cat and the prey does not necessarily mean that these species, especially those weighing >10 kg, are key prey for golden cat. On the contrary, a strong positive spatial association might suggest that these species are neither threatened nor hunted by golden cat. This possibility may be supported by the findings from a similar evergreen rainforest habitat in Taman Negara, Malaysia, which reported the golden cat’s diet to consist of avian body parts, mouse deer, lizards, snakes, rats and langur [19].

There are several possible limitations in our study that we tried to control. Firstly, the camera trap set on the forest floor would not detect arboreal activities of the two felid species. This may include hunting arboreal prey, such as birds and lizards, which camera traps would also not record [19]. Secondly, despite amassing a large data set of independent photographs (>100 for each species) from a substantial sampling effort (28,040 trap nights), only 1.8% of the 13.900 km^2^ Kerinci Seblat National Park was sampled.

Clouded leopards, golden cats and their potential prey species exhibit various activity patterns that indicate the temporal elasticity of both predators. Predators might maximize their hunting effort within prey-rich timing [1, 7]. Similarly, prey might become more active when fewer predators are inactive [1, 3]. Based on these activity patterns there are three possible explanations. First, the spatial niche separation between clouded leopards and golden cats exist, but it was not detected. However, given the high sampling intensity of the study, we judge this to be unlikely. Second, the spatial niche separation between clouded leopards and golden cat does not exist. A considered explanation that niche separation between both cats could be because the mesopredator suppression keeps both species at a density below which competition manifests. However, there is inadequate evidence to support this possibility, and we have not investigated the influence of tigers on these two smaller felids. Third, the interspecific competition between clouded leopards and golden cats is lower due to their temporal separation and the relatively high occurrence of a variety of nocturnal and diurnal prey. Here is the most likely conclusion, considering also that we judge that poaching pressure on these two felids species is low, nevertheless the low occurrence of both cat species in the highly disturbed Ipuh site emphasises the importance of safeguarding forest habitat in Kerinci Seblat landscape.

Our study lends support to calls to not only protect primary rainforest, but also not to disregard the importance of secondary forest to threatened vertebrate communities in the tropics [41, 42]. However, besides ensuring forest integrity and avoiding further degradation, controlling poaching of their ungulate prey will also be a determining factor in the survival of Sumatra’s mesopredators.

## Supporting information

S1 Dataset (ZIP)

S1 Fig Temporal pattern of studied species across study areas. (DOCX)

S1 Table Species photographed during the surveys conducted between April 2014 and December 2016 in the Kerinci Seblat Landscape, Sumatra. (DOCX)

S2 Table List of models with 50,000 iterations and 10,000 burn for the two-species single season occupancy in pooled study areas. (DOCX)

S3 Table Top five occupancy models for focal species, using single season, single species model with the following covariates; elevation, distance to forest edge and distance to river. (DOCX)
