# Supplementary material for: Assessing the spatiotemporal interactions of mesopredators in Sumatra’s tropical rainforest
Source: PLoS One. 2018 Sep 19;13(9):e0202876. doi: 10.1371/journal.pone.0202876 (PMC6145507; doi:10.1371/journal.pone.0202876)
Supplement: S1 Fig — (DOCX) [file pone.0202876.s002.docx]

**S1 Fig. Temporal pattern of studied species across study areas**

| **Species** | **Study Area** | | | |
| --- | --- | --- | --- | --- |
|  | **Bungo** | **Sipurak** | **RKE** | **Ipuh** |
| **C. leopard** | 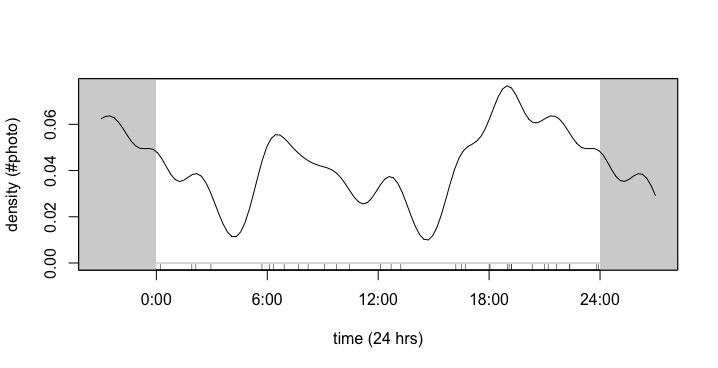 | 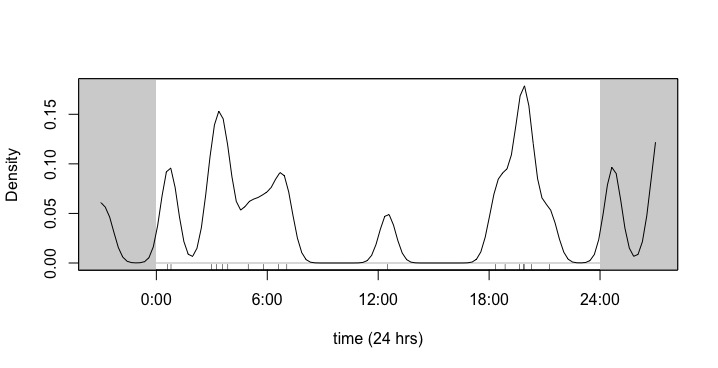 | 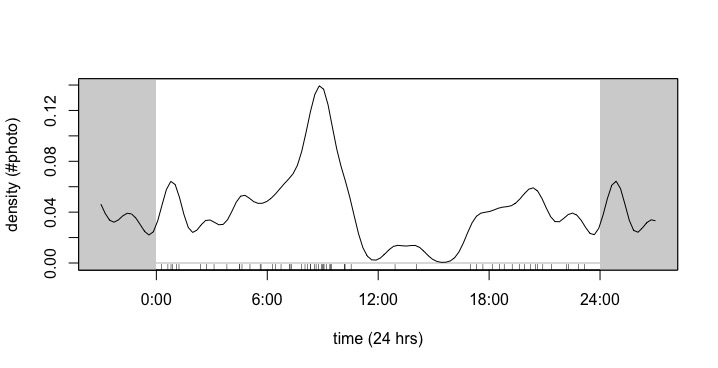 | 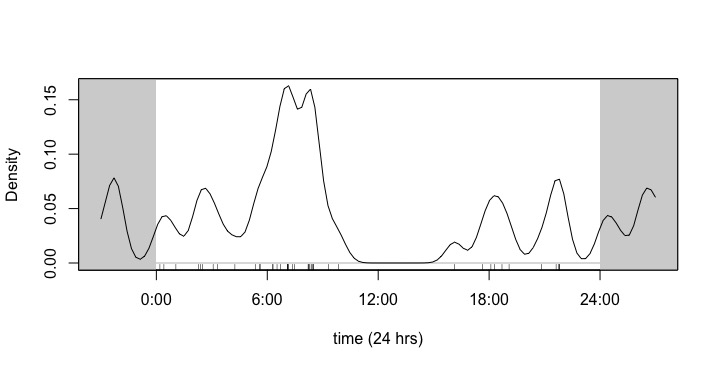 |
| **Golden cat** | 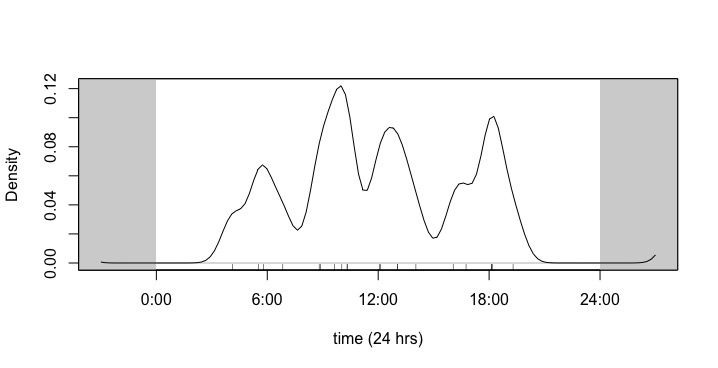 | 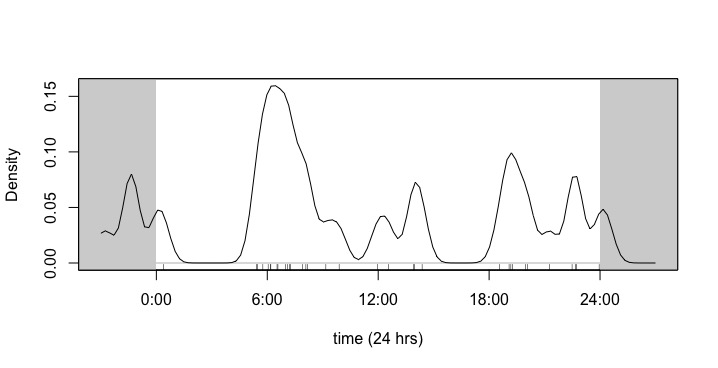 | 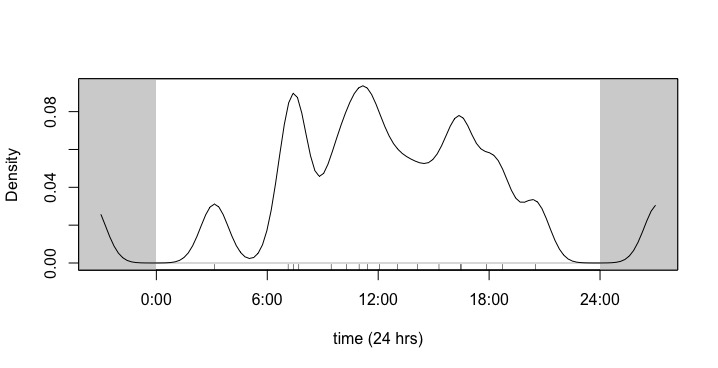 | 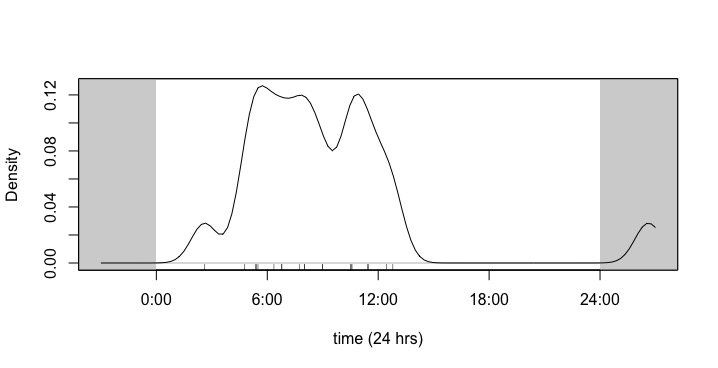 |
| **Muntjac** | 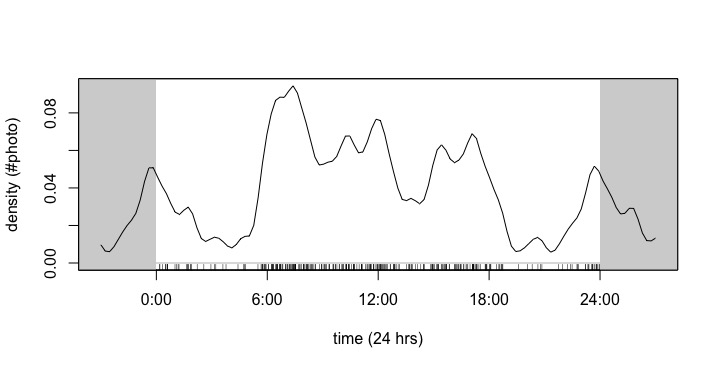 | 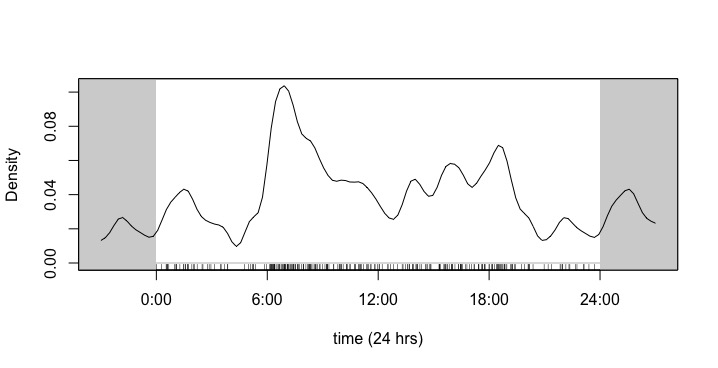 | 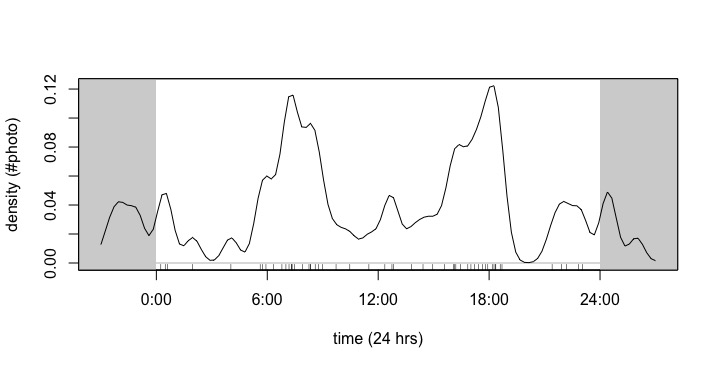 | 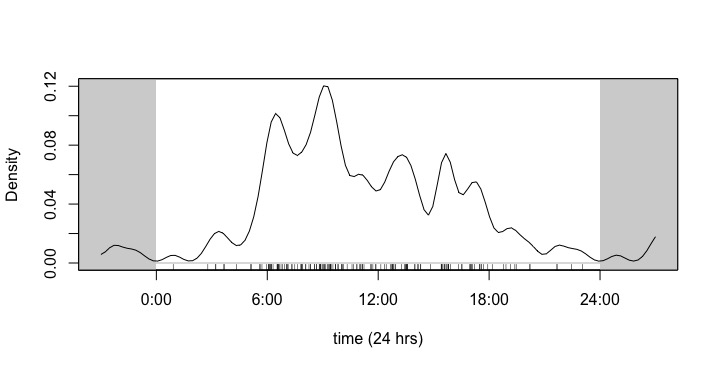 |
| **Mouse deer** | 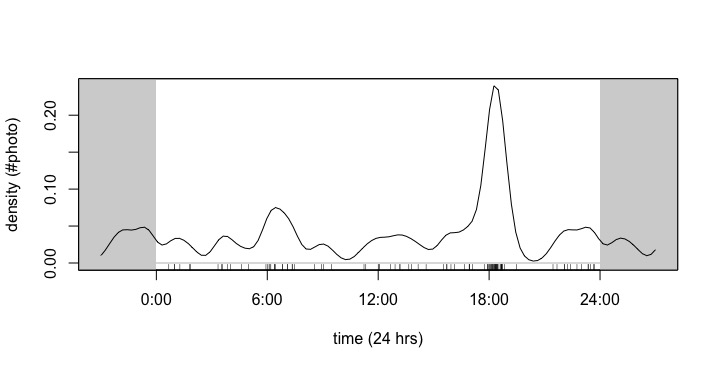 | 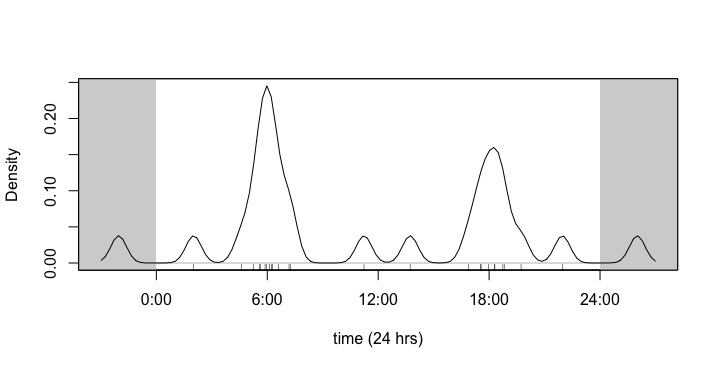 | 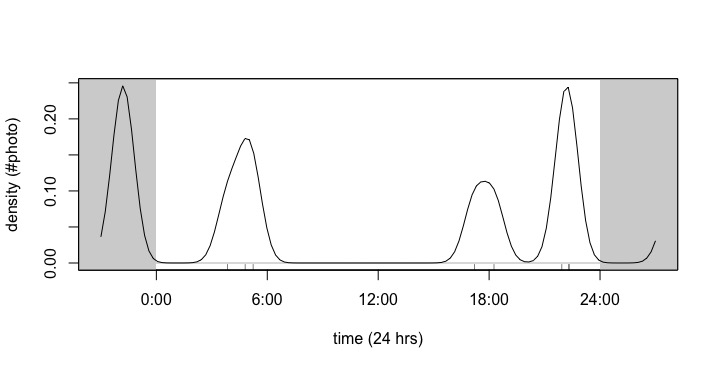 | 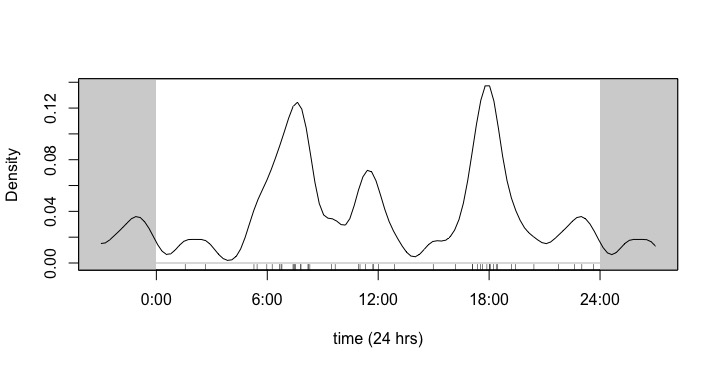 |
| **Macaque** | 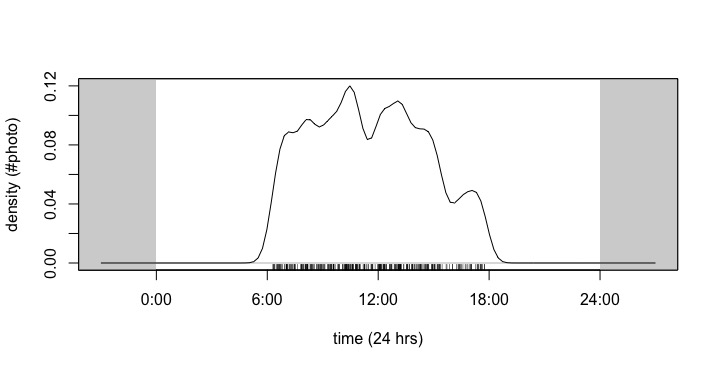 | 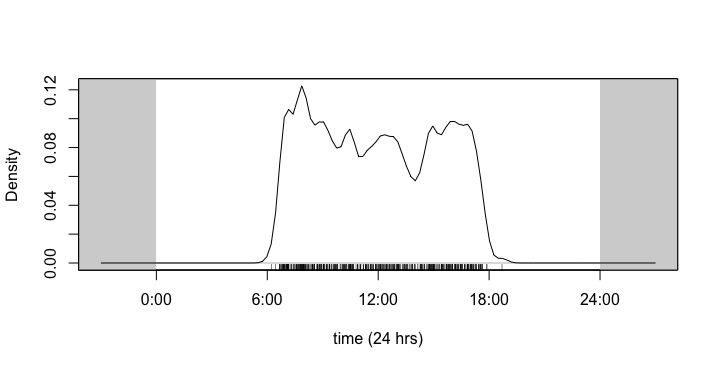 | 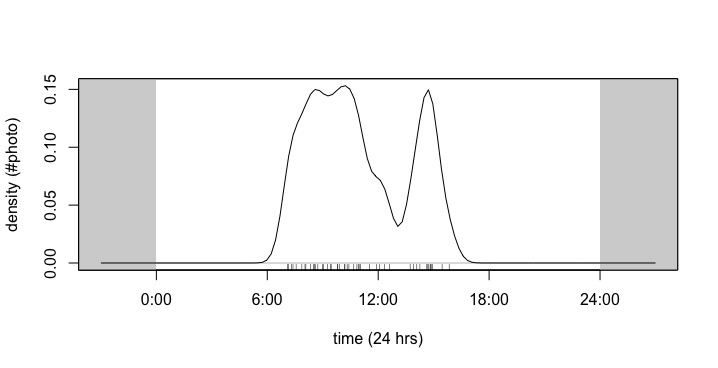 | 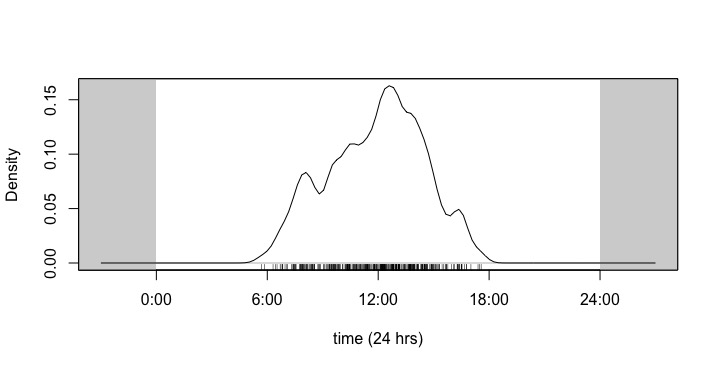 |
| **Porcupine** | 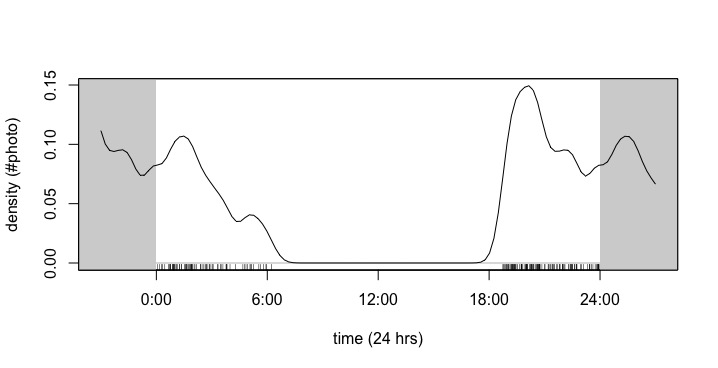 | 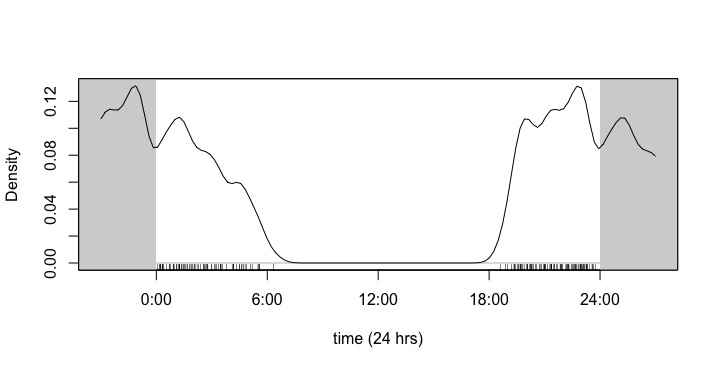 | 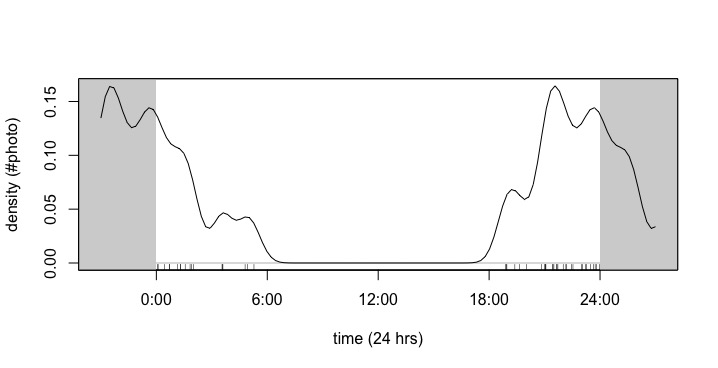 | 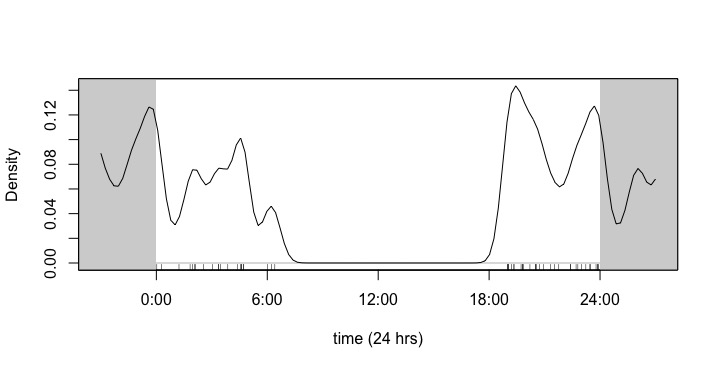 |
| **Great Argus** | 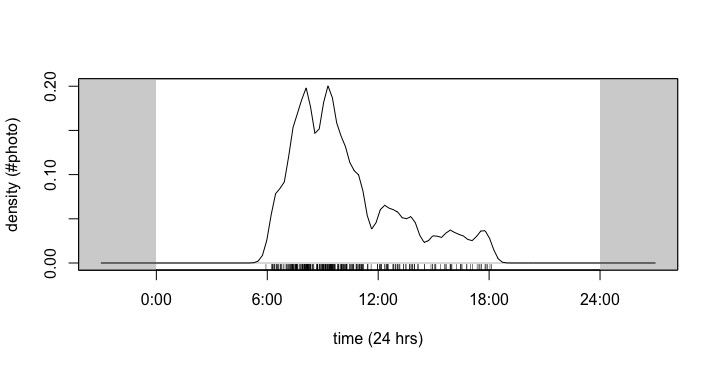 | 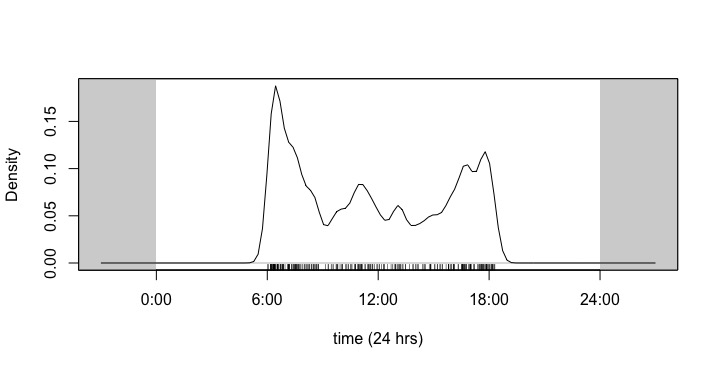 | 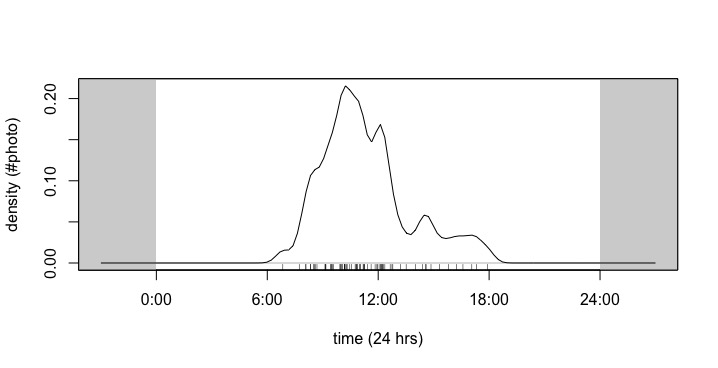 | 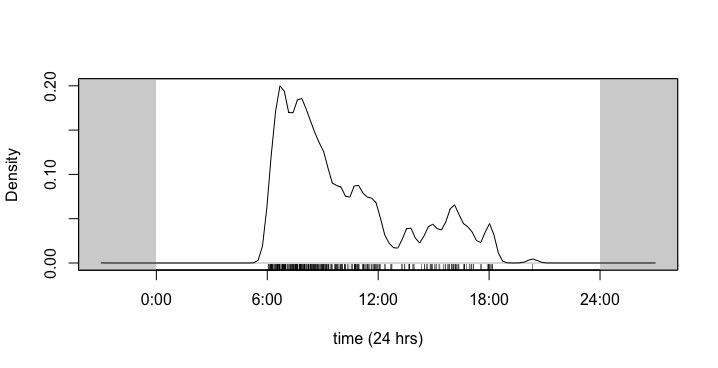 |
