# Supplementary material for: Assessing the spatiotemporal interactions of mesopredators in Sumatra’s tropical rainforest
Source: PLoS One. 2018 Sep 19;13(9):e0202876. doi: 10.1371/journal.pone.0202876 (PMC6145507; doi:10.1371/journal.pone.0202876)
Supplement: S1 Table — (DOCX) [file pone.0202876.s003.docx]

**S1 Table. Species photographed during the surveys conducted between April 2014 and December 2016 in the Kerinci Seblat Landscape, Sumatra.** Note: ∑ Photo is total independent photos of a species but some might be recorded in both cameras, ER is encounter rate, a raw value of total of independent photographs per 100 trapping nights, Naïve ᴪ is a raw value of percentage of ratio of number of camera trap that detected animal and total stations. *) Tragulidae consists two species of greater and lesser mouse deer.

| **No** | **Family** | **IUCN Status** | **English Name**  ***Scientific Name*** | **Bungo** | | | **Sipurak** | | | **RKE** | | | **Ipuh** | | |
| --- | --- | --- | --- | --- | --- | --- | --- | --- | --- | --- | --- | --- | --- | --- | --- |
|  |  |  |  | **∑ Photo** | **ER** | **Naïve ᴪ** | **∑ Photo** | **ER** | **Naïve ᴪ** | **∑ Photo** | **ER** | **Naïve ᴪ** | **∑ Photo** | **ER** | **Naïve ᴪ** |
| 1 | Bovidae | VU | Sumatran serow *Capricornis sumatraensis* | 11 | 0.13 | 6.58 | 11 | 0.16 | 6.58 | 99 | 1.35 | 29.33 | - | - | - |
| 2 | Canidae | EN | Asiatic wild dog *Cuon alpinus* | - | - | - | - | - | - | 21 | 0.21 | 16.00 | 40 | 0.64 | 26.7 |
| 3 | Canidae | n/a | Domestic dog | 2 | 0.02 | 1.32 | 1 | 0.01 | 1.32 | - | - | - | - | - | - |
| 4 | Cercopithecidae | LC | Pig-tailed macaque *Macaca nemestrina* | 413 | 4.92 | 80.26 | 606 | 8.59 | 85.53 | 54 | 0.72 | 29.33 | 373 | 5.94 | 86.7 |
| 5 | Cercopithecidae | NT | Sumatran surili *Presbitis melalophos* | 29 | 0.35 | 10.53 | 2 | 0.03 | 2.63 | 1 | 0.01 | 1.33 | 2 | 0.03 | 1.3 |
| 6 | Cervidae | VU | Sambar *Rusa unicolor* | 12 | 0.14 | 3.95 | 49 | 0.69 | 28.95 | 39 | 0.31 | 1.33 | 9 | 0.14 | 6.7 |
| 7 | Cervidae | LC | Southern red muntjac *Muntiacus muntjak* | 354 | 4.21 | 71.05 | 307 | 4.35 | 81.58 | 60 | 0.67 | 21.33 | 168 | 2.68 | 65.3 |
| 8 | Cervidae | DD | Sumatran mountain muntjac *Muntiacus montanus* | - | - | - | - | - | - | 10 | 0.15 | 2.67 | - | - | - |
| 9 | Felidae | NT | Asiatic golden cat *Pardofelis teminckii* | 22 | 0.26 | 17.11 | 43 | 0.61 | 30.26 | 27 | 0.42 | 21.33 | 20 | 0.32 | 16.0 |
| 10 | Felidae | LC | Leopard cat *Prinolurus benghalensis* | 1 | 0.01 | 1.32 | - | - | - | - | - | - | 1 | 0.02 | 1.3 |
| 11 | Felidae | VU | Marbled cat *Pardofelis marmorata* | 5 | 0.06 | 5.26 | 22 | 0.31 | 17.11 | 6 | 0.09 | 4.00 | 11 | 0.18 | 10.7 |
| 12 | Felidae | CR | Sumatran tiger *Panthera tigris sumantrae* | 36 | 0.43 | 25.00 | 97 | 1.38 | 40.79 | 2 | 0.03 | 2.67 | 17 | 0.27 | 13.3 |
| 13 | Felidae | VU | Sunda clouded leopard *Neofelis diardi* | 35 | 0.42 | 23.68 | 24 | 0.34 | 14.47 | 65 | 0.93 | 34.67 | 44 | 0.70 | 21.3 |
| 14 | Hystricidae | LC | Malayan porcupine *Histryx brachyura* | 208 | 2.48 | 50.00 | 232 | 3.29 | 50.00 | 60 | 0.73 | 21.33 | 54 | 0.86 | 24.0 |
| 15 | Leporidae | VU | Sumatran rabbit *Nesolagus netscheri* | - | - | - | - | - | - | 1 | 0.01 | 1.33 | - | - | - |
| 16 | Manidae | CR | Sunda pangolin *Manis javanica* | 4 | 0.05 | 5.26 | 5 | 0.07 | 6.58 | - | - | - | 2 | 0.03 | 1.3 |
| 17 | Mustelidae | NT | Hog badger *Arctonyx hoevenii* | 2 | 0.02 | 1.32 | - | - | - | 1 | 0.01 | 1.33 | - | - | - |
| 18 | Mustelidae | LC | Yellow throated marten *Martes flavigula* | - | - | - | 30 | 0.43 | 21.05 | 21 | 0.31 | 12.00 | 10 | 0.16 | 12.0 |
| 19 | Phasianidae | LC | Bronze-tailed peacock-pheasant  *Polyplectron chalcurum* | - | - | - | 6 | 0.09 | 2.63 | 9 | 0.10 | 4.00 | - | - | - |
| 20 | Phasianidae | NT | Great argus *Argussianus argus* | 450 | 5.36 | 47.37 | 414 | 5.87 | 43.42 | 123 | 1.12 | 20.00 | 347 | 5.53 | 70.7 |
| 21 | Phasianidae | LC | Red junglefowl *Gallus gallus* | 1 | 0.01 | 1.32 | 4 | 0.06 | 2.63 | 1 | 0.10 | 1.33 | - | - | - |
| 22 | Phasianidae | LC | Red-billed partridge  *Arborophila rubrirostris* | - | - | - | 3 | 0.04 | 3.95 | 4 | 0.04 | 2.67 | 13 | 0.21 | 2.7 |
| 23 | Phasianidae | NT | Salvadori's Pheasant *Lophura inornata* | 1 | 0.01 | 1.32 | 2 | 0.03 | 2.63 | 18 | 0.27 | 13.33 | 5 | 0.08 | 5.3 |
| 24 | Prionodontidae | LC | Banded linsang *Prionodon linsang* | 7 | 0.08 | 3.95 | 26 | 0.37 | 13.16 | 16 | 0.24 | 9.33 | 5 | 0.08 | 5.3 |
| 25 | Pteropopidae | VU | Sunda fruit bat *Acerodon mackloti* | 50 | 0.60 | 26.32 | - | - | - | - | - | - | - | - | - |
| 26 | Suidae | LC | Bearded pig *Sus barbatus* | - | - | - | 1 | 0.01 | 1.32 | 100 | 0.94 | 13.33 | 294 | 4.68 | 60.0 |
| 27 | Suidae | LC | Wild boar *Sus scrofa* | 39 | 0.46 | 15.79 | 32 | 0.45 | 19.74 | 43 | 0.49 | 20.00 | 73 | 1.16 | 45.3 |
| 28 | Tapiridae | EN | Malayan tapir *Tapirus indicus* | 142 | 1.69 | 42.11 | 90 | 1.28 | 40.79 | 68 | 0.84 | 36.00 | 79 | 1.26 | 42.7 |
| 29 | Tragulidae* | LC | Mousedeer *Tragulus spp.* | 127 | 1.51 | 25.00 | 29 | 0.41 | 17.11 | 8 | 0.12 | 2.67 | 48 | 0.76 | 26.7 |
| 30 | Tupaiidae | LC | Large tree shrew *Tupaia tana* | 8 | 0.10 | 7.89 | 9 | 0.13 | 10.53 | - | - | - | 15 | 0.24 | 9.3 |
| 31 | Tupaiidae | LC | Common treeshrew *Tupaia glis* | - | - | - | - | - | - | 11 | 0.16 | 10.67 | - | - | - |
| 32 | Turdidae | LC | Shiny Whistling-thrush  *Myophonus melanurus* | - | - | - | - | - | - | 2 | 0.03 | 1.33 | - | - | - |
| 33 | Ursidae | VU | Malayan sun bear *Helarctos malayanus* | 116 | 1.38 | 46.05 | 79 | 1.12 | 35.53 | 18 | 0.21 | 14.67 | 120 | 1.91 | 53.3 |
| 34 | Varanidae | LC | Monitor lizard *Varanus salvathor* | 1 | 0.01 | 1.32 | - | - | - | - | - | - | - | - | - |
| 35 | Viverridae | VU | Banded civet *Hemigalus derdyanus* | - | - | - | - | - | - | 7 | 0.10 | 2.67 | 10 | 0.16 | 8.0 |
| 36 | Viverridae | VU | Binturong *Arctictis binturong* | 6 | 0.07 | 6.58 | 5 | 0.07 | 6.58 | - | - | - | 4 | 0.06 | 5.3 |
| 37 | Viverridae | LC | Common palm civet  *Paradoxurus hermaphroditus* | - | - | - | - | - | - | 82 | 1.18 | 21.33 | 1 | 0.02 | 1.3 |
| 38 | Viverridae | LC | Masked palm civet *Paguma larvata* | 35 | 0.42 | 18.42 | 42 | 0.60 | 18.42 | - | - | - | - | - | - |
| 39 | Viverridae | LC | Small-toothed palm civet  *Arctogalidia trivirgata* | 1 | 0.01 | 1.32 | - | - | - | - | - | - | - | - | - |
| 40 | n/a | n/a | Unknown people/trespassers | 72 | 0.86 | 13.16 | 42 | 0.60 | 27.63 | 6 | 0.07 | 4.00 | 79 | 1.26 | 26.7 |
