# Supplementary material for: Assessing the spatiotemporal interactions of mesopredators in Sumatra’s tropical rainforest
Source: PLoS One. 2018 Sep 19;13(9):e0202876. doi: 10.1371/journal.pone.0202876 (PMC6145507; doi:10.1371/journal.pone.0202876)
Supplement: S2 Table — (DOCX) [file pone.0202876.s004.docx]

**S2 Table. List of models with 50,000 iterations and 10,000 burn in for the two-species single season occupancy in pooled study areas. Note:** *grey cells indicate significance

| **Value** | **Species-relation** | **JAGS Parameters** | **Mean** | **(SD)** | **HDIlo** | **HDIup** | **R** |
| --- | --- | --- | --- | --- | --- | --- | --- |
| Β Coefficient of site covariates | Argus-elevation | beta.elev.psi.ar | -1.371 | 0.225 | -1.848 | -0.973 | 1.001 |
|  | Golden cat-elevation | beta.elev.psi.gc | 2.631 | 1.242 | 0.426 | 5.446 | 1.001 |
|  | Macaque-elevation | beta.elev.psi.mc | -1.575 | 0.359 | -2.354 | -1.001 | 1.001 |
|  | Porcupine-elevation | beta.elev.psi.pc | -0.326 | 0.139 | -0.603 | -0.065 | 1.001 |
|  | Argus-forest edge | beta.forest.psi.ar | 0.282 | 0.156 | -0.024 | 0.589 | 1.001 |
|  | Clouded leopard-forest edge | beta.forest.psi.cl | 0.307 | 0.242 | -0.156 | 0.795 | 1.004 |
|  | Muntjac-forest edge | beta.forest.psi.mj | 0.547 | 0.153 | 0.257 | 0.855 | 1.001 |
|  | Argus-distance to river | beta.river.psi.ar | -0.343 | 0.152 | -0.643 | -0.049 | 1.001 |
|  | Golden cat-distance to river | beta.river.psi.gc | -1.991 | 1.736 | -6.283 | 0.493 | 1.010 |
| Two-species occupancy | Argus-clouded leopard | beta.psi.WithAR.cl | -0.391 | 0.466 | -1.360 | 0.523 | 1.002 |
|  | Argus-golden cat | beta.psi.WithAR.gc | 1.665 | 2.528 | -2.612 | 8.135 | 1.004 |
|  | Clouded leopard-golden cat | beta.psi.WithCL.gc | 6.328 | 2.024 | 2.390 | 9.755 | 1.004 |
|  | Macaque-clouded leopard | beta.psi.WithMC.cl | -1.567 | 0.652 | -3.027 | -0.476 | 1.005 |
|  | Macaque-golden cat | beta.psi.WithMC.gc | 4.913 | 2.902 | -2.147 | 9.567 | 1.009 |
|  | Mousedeer-clouded leopard | beta.psi.WithMD.cl | -1.488 | 0.683 | -2.888 | -0.264 | 1.002 |
|  | Mousedeer-golden cat | beta.psi.WithMD.gc | 6.775 | 2.283 | 1.839 | 9.880 | 1.002 |
|  | Muntjac-clouded leopard | beta.psi.WithMJ.cl | -0.526 | 0.521 | -1.529 | 0.525 | 1.001 |
|  | Muntjac-golden cat | beta.psi.WithMJ.gc | -1.417 | 3.045 | -7.828 | 4.501 | 1.018 |
|  | Porcupine-clouded leopard | beta.psi.WithPC.cl | 1.497 | 0.654 | 0.452 | 2.981 | 1.002 |
|  | Porcupine-golden cat | beta.psi.WithPC.gc | 3.717 | 2.994 | -1.809 | 9.492 | 1.002 |
|  |  | deviance | 7862.793 | 62.62 | 7744.285 | 7986.396 | 1.003 |
