# Supplementary material for: Assessing the spatiotemporal interactions of mesopredators in Sumatra’s tropical rainforest
Source: PLoS One. 2018 Sep 19;13(9):e0202876. doi: 10.1371/journal.pone.0202876 (PMC6145507; doi:10.1371/journal.pone.0202876)
Supplement: S3 Table — (DOCX) [file pone.0202876.s005.docx]

**S3 Table. Top five occupancy models for focal species, using single season, single species model with the following covariates; elevation, distance to forest edge and distance to river.** Note: K is number of parameters, AICc is Akaike Information Criterion corrected for small sample size, ∆AICc/ delta AICc indicates difference value of a model from the best candidate model, and W_i_ is Akaike weight, the bigger indicate the model is more plausible.

| **ID** | **Model** | ***K*** | **AICc** | **∆ AICc** | ***W_i_*** |
| --- | --- | --- | --- | --- | --- |
| *Clouded leopard* | |  |  |  |  |
| 1.1 | psi(studyarea+forest),p(.) | 6 | 854.08 | 0.00 | 0.45 |
| 1.2 | psi(studyarea+forest+river),p(.) | 7 | 855.62 | 1.54 | 0.21 |
| 1.3 | psi(studyarea+elev),p(.) | 6 | 856.77 | 2.70 | 0.12 |
| 1.4 | psi(studyarea+elev+forest+river),p(.) | 8 | 857.51 | 3.43 | 0.08 |
| 1.5 | psi(studyarea),p(.) | 5 | 857.91 | 3.83 | 0.07 |
|  |  |  |  |  |  |
| *Golden cat* | |  |  |  |  |
| 2.1 | psi(elev),p(.) | 3 | 705.12 | 0.00 | 0.23 |
| 2.2 | psi(elev+river),p(.) | 4 | 705.82 | 0.70 | 0.16 |
| 2.3 | psi(studyarea),p(.) | 5 | 706.46 | 1.34 | 0.12 |
| 2.4 | psi(elev+forest),p(.) | 4 | 706.72 | 1.60 | 0.10 |
| 2.5 | psi(.),p(.) | 2 | 707.33 | 2.21 | 0.08 |
|  |  |  |  |  |  |
| *Muntjac* | |  |  |  |  |
| 3.1 | psi(studyarea),p(.) | 5 | 1968.16 | 0.00 | 0.28 |
| 3.2 | psi(studyarea+forest),p(.) | 6 | 1968.28 | 0.12 | 0.27 |
| 3.3 | psi(studyarea+elev),p(.) | 6 | 1969.78 | 1.62 | 0.13 |
| 3.4 | psi(studyarea+elev+forest+river),p(.) | 8 | 1969.92 | 1.77 | 0.12 |
| 3.5 | psi(studyarea+river),p(.) | 6 | 1970.13 | 1.98 | 0.11 |
|  |  |  |  |  |  |
| *Mouse deer* | |  |  |  |  |
| 4.1 | psi(.),p(.) | 2 | 669.81 | 0.00 | 1.00 |
| 4.2 | psi(studyarea),p(.) | 5 | 721.86 | 52.05 | 0.00 |
| 4.3 | psi(studyarea+forest),p(.) | 6 | 723.43 | 53.62 | 0.00 |
| 4.4 | psi(studyarea+river),p(.) | 6 | 723.94 | 54.13 | 0.00 |
| 4.5 | psi(studyarea+elev),p(.) | 6 | 723.95 | 54.14 | 0.00 |
|  |  |  |  |  |  |
| *Macaque* | |  |  |  |  |
| 5.1 | psi(studyarea+elev),p(.) | 6 | 2156.82 | 0.00 | 0.69 |
| 5.2 | psi(studyarea+elev+forest+river),p(.) | 8 | 2158.45 | 1.63 | 0.31 |
| 5.3 | psi(elev+forest),p(.) | 4 | 2166.83 | 10.01 | 0.01 |
| 5.4 | psi(studyarea),p(.) | 5 | 2171.08 | 14.26 | 0.00 |
| 5.5 | psi(studyarea+forest),p(.) | 6 | 2172.46 | 15.64 | 0.00 |
|  |  |  |  |  |  |
| *Porcupine* | |  |  |  |  |
| 6.1 | psi(studyarea+elev),p(.) | 6 | 1314.84 | 0.00 | 0.70 |
| 6.2 | psi(studyarea+elev+forest+river),p(.) | 8 | 1316.65 | 1.81 | 0.28 |
| 6.3 | psi(studyarea+forest),p(.) | 6 | 1322.63 | 7.79 | 0.01 |
| 6.4 | psi(studyarea+forest+river),p(.) | 7 | 1324.65 | 9.81 | 0.01 |
| 6.5 | psi(.),p(.) | 2 | 1325.72 | 10.88 | 0.00 |
|  |  |  |  |  |  |
| *Argus* | |  |  |  |  |
| 7.1 | psi(studyarea+elev+forest+river),p(.) | 8 | 1624.54 | 0.00 | 0.73 |
| 7.2 | psi(elev+river),p(.) | 4 | 1627.74 | 3.19 | 0.15 |
| 7.3 | psi(elev+forest),p(.) | 4 | 1629.45 | 4.91 | 0.06 |
| 7.4 | psi(elev),p(.) | 3 | 1630.56 | 6.01 | 0.04 |
| 7.5 | psi(studyarea+elev),p(.) | 6 | 1631.78 | 7.24 | 0.02 |
